# The Premium of Public Perceived Greenery: A Framework Using Multiscale GWR and Deep Learning

**DOI:** 10.3390/ijerph18136809

**Published:** 2021-06-24

**Authors:** Yonglin Zhang, Xiao Fu, Chencan Lv, Shanlin Li

**Affiliations:** 1 State Key Laboratory of Urban and Regional Ecology, Research Center for Eco-Environmental Sciences, Chinese Academy of Sciences, Beijing 100085, China; ylzhang@rcees.ac.cn (Y.Z.); xiaofu@rcees.ac.cn (X.F.); cclv_st@rcees.ac.cn (C.L.); 2 National Science Library, Chinese Academy of Sciences, Beijing 100190, China

**Keywords:** multiscale GWR (MGWR), big geodata, deep learning, hedonic price model, housing premium, public perceived greenery

## Abstract

Population agglomeration and real estate development encroach on public green spaces, threatening human settlement equity and perceptual experience. Perceived greenery is a vital interface for residents to interact with the urban eco-environment. Nevertheless, the economic premiums and spatial scale of such greenery have not been fully studied because a comprehensive quantitative framework is difficult to obtain. Here, taking advantage of big geodata and deep learning to quantify public perceived greenery, we integrate a multiscale GWR (MGWR) and a hedonic price model (HPM) and propose an analytic framework to explore the premium of perceived greenery and its spatial pattern at the neighborhood scale. Our empirical study in Beijing demonstrated that (1) MGWR-based HPM can lead to good performance and increase understanding of the spatial premium effect of perceived greenery; (2) for every 1% increase in neighborhood-level perceived greenery, economic premiums increase by 4.1% (115,862 RMB) on average; and (3) the premium of perceived greenery is spatially imbalanced and linearly decreases with location, which is caused by Beijing’s monocentric development pattern. Our framework provides analytical tools for measuring and mapping the capitalization of perceived greenery. Furthermore, the empirical results can provide positive implications for establishing equitable housing policies and livable neighborhoods.

## 1. Introduction

As a vital component of the urban landscape, urban green space provides security for the coordinated development of social-economic-natural complex ecosystems [1]. Urban parks and forests provide essential ecological benefits in maintaining biodiversity and performing carbon fixation [2,3]. Some scattered green spaces, such as road greenery [4] and green roofs [5], can regulate the microclimate [6,7], block noise [8], and reduce greenhouse gases [9]. From the perspective of social service functions, urban greenery provides residents with areas for leisure and entertainment, outdoor exercise, social activities [10,11,12], and psychological stress relief [13,14]; such areas also provide aesthetic beauty. In recent years, population agglomeration and real estate development have extruded public green spaces, threatening the livable human environment and perceived greenery equity. Recently, the economic premiums and equity of urban greenery have attracted the attention from many fields, such as urban ecology, design, planning, and real estate economics [15,16,17,18,19,20]. Deeply studying the large-scale economic benefits of urban greenery is of great value for sustainable urban development and equitable living conditions.

Public perceived greenery can be described as residents’ visual exposure to urban green spaces and ornamental forestland [21,22]. As a well-recognized amenity, pleasant perceived greenery can be capitalized into residential and commercial property values [23,24,25]. For instance, a case study in Salo, Finland, showed that housing with forest landscapes is 4.9% more expensive than regular housing [26]. Jim and Chen [27] showed that a pleasant green view in Guangzhou, China, positively affects housing prices, with a premium rate of 6.9%. A case study in Singapore showed that tropical managed vegetation had a positive premium on 98% of property prices, representing 3% of the average property value [28]. Recently, several empirical studies have integrated new techniques into the study of urban greenery premiums. Kang et al. [29] used a machine learning (ML) approach to predict the housing appreciation rate in Boston, USA. Simultaneously, Li, et al. [30] adopted an ML-based algorithm to understand the major influential factors and their contributions in Shenzhen, China. Chen, et al. [31] further explored the effect of environmental factors on housing prices based on ensemble learning and indicated that there was a positive effect when the greening level reached a certain level. However, the previous studies neglected the spatial premium scales and variation of public perceived greenery. There is a lack of suitable means to explain the spatial variation of the influential factors in built environments. The contribution of perceived greenery to the housing premium, as well as its spatial pattern and impact scales, need further interpretation and discussion. Understanding the mechanisms influencing the spatial premium of such greenery is essential to optimize the spatial layout, equalize public resources, and formulate sustainable development policies.

Although the hedonic price model (HPM) or the HPM-based frameworks have been widely adopted to housing premium studies and can identify the marginal prices of specific influential factors [27,32,33], there are still deficiencies in greenery-focused HPM studies: (1) difficulty in depicting the spatial premium patterns of influential factors and their various impact scales and (2) a lack of large-scale sensing methods for public greenery at the neighborhood scale. Therefore, the aim of this study is to refine HPM by integrating novel big geodata, deep learning approaches, and multiscale spatial models into a comprehensive analytical framework. The integrated framework was adopted to improve the understanding of the quantitative connections between public perceived greenery and economic premiums in large-scale scenarios, and to provide a reference for governments to establish equitable housing policies and livable neighborhoods.

This article is organized as follows. Section 2 includes five main parts: (1) the study area, (2) a comprehensive analytical framework, (3) deep learning for perceived greenery measurement, and (4) the formulas of the models, variable definitions, and statistics. Section 3 introduces the experimental results, compares the model performance in detail, explains the main findings, and provides policy recommendations.

### 1.1. Measuring Large-Scale Perceived Greenery Using Street Views and Deep Learning

Traditional means of quantifying perceived greenery, such as satellite remote sensing images and aerial photography, differ greatly from the human perception perspective and field of view [32], which makes it difficult to characterize physical setting perceptions. In traditional visual landscape surveys, field photography is often adopted to obtain scenario photos [33]. Then, a visual interpretation is used to quantify perceived greenery [34]. In recent years, deep learning and big geodata approaches have led to new insights into the quantification of urban perception globally [35,36]. Big geodata provide a reliable source for exploring human perceived greenery [35]. As a typical geotagged data source, street views provide excellent spatial coverage, accuracy, and costs, and are widely adopted in studying the physical configuration and social perception of cities [37,38,39,40]. Zhang and Dong [37] and Ye et al. [39] showed that the street-level greenery of Beijing and Shanghai both have an enhancing effect on house prices. In addition, the combination of streetscape images and computer vision can solve data magnitude and coverage problems [41]. Dong et al. [42] measured the street-level greenery in Beijing’s central area based on computer vision and found that there is a serious imbalance in the public perception of road greenery through spatial clustering. Fu et al. [38] mapped street greenery using the PSPNet [43] deep learning framework. The comparison found that the average greening quality in Shanghai was better than that in Beijing. However, the previous HPM analytical frameworks for neighborhood-level perceived greenery quantification are still immature [37,38]. In this paper, massive street views and deep learning are used to quantify the neighborhood-level perceived greenery around housing estates to provide a quantitative metric for economic premium analysis at a large scale. 

### 1.2. A Novel Hedonic Price Modeling Framework Based on MGWR

The HPM is a classic method used to quantify and explain the characteristics that affect the value of public goods. This approach was mainly derived from Rosen’s [44] implicit price theory and Lancaster’s [45] heterogeneous consumption theory, which assess marginal premiums by measuring people’s willingness to pay. According to consumer theory [45], the satisfaction of the utility associated with a unit characteristic quantity varies for different consumers. In the secondhand housing market, for example, economic premiums can largely reflect the buyer’s subjective preference for various characteristics.

Previous studies have often employed the ordinary least squares (OLS) method to construct HPMs [37,38,39] but ignored the spatial heterogeneity of economic premiums. The OLS-based HPM assumes that all housing characteristics are homogenous and mutually independent, but these assumptions differ highly from actual conditions [46]; empirical studies have shown that geographically weighted regression (GWR) [47] is more suitable for explaining housing characteristics and their spatial heterogeneity [48]. The premiums of housing characteristics may appear spatially stationary or nonstationary, but the OLS-based model ignores spatial variations, which may cause the coefficient estimates to be biased [49,50].

A fixed bandwidth is adopted in GWR to determine the boundary of local regression [47] and approximately explain the scales of spatial processes. The GWR-based HPM assumes that all housing characteristics have the same spatial scale; however, the scales of spatial processes are usually heterogeneous [46,47]. Recently, Fotheringham et al. [51] proposed the multiscale GWR (MGWR) approach in the field of spatial econometrics, which has the following advantages. First, it allows each covariate to have an independent bandwidth. Second, the multi-bandwidth approach allows MGWR to reflect actual conditions, thereby facilitating the interpretation of variables [51,52]. In summary, this study employs MGWR as an improved form of the HPM to analyze the spatial premium effect of public perceived greenery in detail.

## 2. Materials and Methods

### 2.1. Study Area

Beijing has the top financial, scientific, and educational resources in China, and it has considerable national and international influence. The agglomeration of talent and migrants has led to fierce competition for settlement in Beijing. With residential land use approaching saturation, secondhand transactions have become the driver of the real estate market. At the end of 2020, the average house price in Beijing reached 60,143 RMB per square meter.

**Figure 1:**
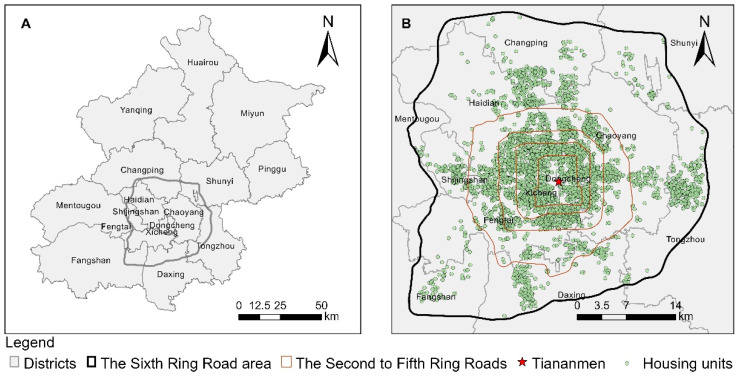
The Sixth Ring Road area and housing units in Beijing. Subplot (**A**): the location of study area. The red lines from the inside to the outside in subplot (**B**) are the Second to Fifth Ring Roads.

The built-up areas in Beijing have expanded rapidly for nearly half a century, and they are divided by five major motorways from the inside to the outside (see Figure 1), forming a typical monocentric ring layout. The length of the centerline of the road network in the study area is 5640 km, and the main built-up area is concentrated in the Sixth Ring Road (referred to as the sixth ring area for short), with a total area of 2265 km^2^. The Sixth Ring Road area encompasses the working and living space of most residents in Beijing, and the housing prices in different locations vary significantly [37,38]. The government must address the balance between real estate development and greening configuration in high-density and high-population areas.

### 2.2. Analytical Framework

**Figure 2:**
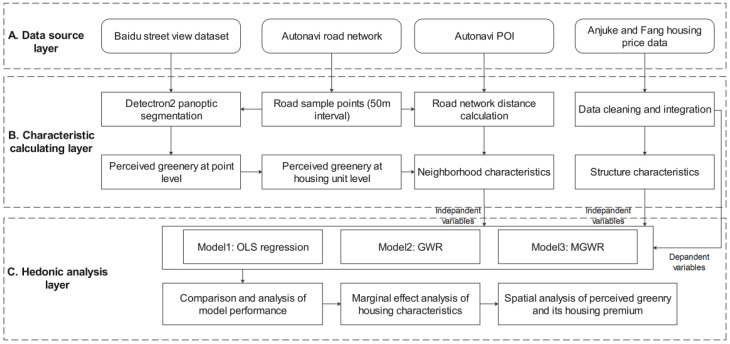
The analytical framework.

This article proposes a three-layer framework (see Figure 2) that includes data sources, characteristic calculations, and hedonic analysis layers. The data source layer includes Baidu street views, AutoNavi road network data, AutoNavi points of interest (POIs), and housing price data (Anjuke and Fang platforms’ transaction records in 2015). The characteristic calculation layer includes the generation of equidistant samples of the road network, panoptic segmentation, perceived greenery and accessibility indicator calculations, and the cleaning and integration of housing data. In addition, to address multisource data fusion, the neighborhood and structural indicators are all aggregated to the neighborhood level. In the hedonic analysis layer, we first compare the performance and parameter estimates of the OLS, GWR, and MGWR models; analyze the capitalization of house characteristics based on HPM theory; and focus on the premium of perceived greenery and spatial analysis.

The tools involved in the framework include (1) MGWR 2.2 software (Arizona State University, Tempe, AZ, USA) [52] for OLS, GWR, and MGWR modeling and diagnosis; (2) ArcGIS 10.8 (Environmental Systems Research Institute, Inc., Redlands, CA, USA) for road network accessibility analysis, map visualization, and multisource data fusion; and (3) Python scripts (version 3.8) (Python Software Foundation, www.python.org, accessed on 23 June 2021) for data cleaning and exploratory analysis.

### 2.3. The Calculation of Neighborhood-Level Perceived Greenery

We employed the panoptic segmentation module to calculate the perceptual proportion of greenery (trees, shrubs, and grass). Perceived greening can be defined as the overall visual contact intensity of urban greenery in an in-situ scene. The corresponding formula is as follows:

(1) $$PG=\frac{\sum_{i=1}^{n}Area_{greenry}}{\sum_{i=1}^{n}Area_{total}}\times 100\%$$

where the numerator represents the total area of the street view corresponding to each sample point (including four horizontal perspectives: east, south, west, and north) and the denominator represents the area of greenery masked by panoptic segmentation.

We obtained 110,812 road network sample points at intervals of 50 m based on the method of Dong et al. [42]. To match the dates of the street view and housing data and to consider the deciduous period of trees in the study area, the results obtained from April to October 2015 were selected from the database (78,127 effective points). In the model training process, we used the ADE20K dataset [53] to train a deep convolutional neural network (DCNN), and the overall classification accuracy at the pixel level reached 77.36%. Finally, we used Formula (1) to calculate the street view scores for all sample points, and the results are summarized in a table.

**Figure 3:**
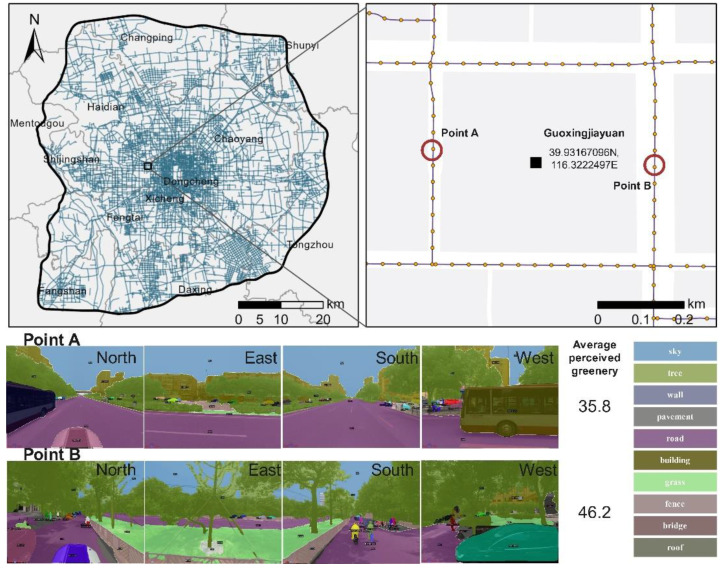
The location of the Guoxingjiayuan community and perceived greenery measurement examples. The vermilion circles in the enlarged map indicate points A and B adjacent to the plot and the corresponding street views used in panoptic segmentation masks.

The sample points around blocks with housing units were used to measure the neighborhood-scale perceived greenery (an 80 m border buffer). On average, there were approximately 23 points in each plot involved in the calculation. Taking the Guoxingjiayuan community in Beijing as an example, two streetscapes with classification masks are shown in Figure 3. The average perceived greenery scores at points A and B were 35.8 and 46.2, respectively.

### 2.4. Hedonic Price Models (HPMs) and Characteristic Statistics

According to HPM theory [44,45], secondhand housing, as a heterogeneous public good, is associated with various characteristic attributes. The impact of a characteristic on price is called a marginal premium. The HPM often determines premiums through a regression equation. Its basic form is as follows:

(2) $$HP=f\left(C_{1},\;C_{2},\ldots ,C_{n}\right)$$

where $C_{n}$ represents the number i of characteristics and HP is the average price of a housing unit. The HPM generally has three forms: linear, logarithmic, and double-logarithmic. This article uses a logarithmic model for modeling. According to HPM theory, the logarithmic model can explain the percentage change in housing premiums associated with a one-unit change in the independent variable when other variables are fixed.

#### 2.4.1. The Ordinary Least Square (OLS) Model

The OLS method expresses the HPM in a linear form and sums the various characteristic factors. The formula is as follows:

(3) $$HP=\sum^{\;}\beta_{i}C_{i}+\varepsilon$$

where ε is the error term, $\beta_{i}$ is the nonstandardized coefficient of the ith feature, and $C_{i}$ is the ith characteristic.

#### 2.4.2. The Geographical Weighted Regression (GWR) Model

The GWR model is based on locally weighted regression and variable parameters, and it embeds spatial position into the regression parameter set on a sample-by-sample basis. The representation of the HPM is as follows:

(4) $$HP_{j}=\sum_{j}\beta_{j}\left(u_{i},\;v_{i}\right)C_{ij}+\varepsilon_{i}$$

where $\left(u_{i},\;v_{i}\right)$ are the coordinates of the ith sample, $C_{ij}$ is the value of the jth characteristic of the ith sample, and $\varepsilon_{i}$ is the error term for the ith sample.

#### 2.4.3. The Multiscale GWR Model

Multiscale GWR (MGWR) further relaxes the experimental assumptions, allowing the relationships between the dependent variable and different independent variables to vary at different scales (bandwidths). The corresponding expression is as follows:

(5) $$HP_{j}=\sum_{j}\beta_{bwj}\left(u_{i},\;v_{i}\right)C_{ij}+\varepsilon_{i}$$

where bwj represents the bandwidth used by the coefficient of the jth variable. MGWR uses a back-fitting algorithm to fit each smoothing term and uses the gold section method to search for the optimal bandwidth by default. The adaptive quadratic kernel function is used for iterative correction. The formula is as follows:

(6) $$w_{ij}=\{\begin{array}{c} {\left[1-{\left[d_{ij}/g_{i}\right]}^{2}\right]}^{2},if\;d_{ij}<g_{i}\\ 0\;\;\;\;\;\;\;\;\;\;\;\;\;,\;otherwise \end{array}$$

where $d_{ij}$ is the distance between sample points i and j and $g_{i}$ is the distance between sample point i and neighbor *K*. *K* is the optimal number of nearest neighbors, which is determined by the corrected Akaike information criterion (AICc). Its formula is as follows:

(7) $$AICc=2n\ln\left(\hat{\sigma }\right)+n\ln\left(2\pi \right)+n\left(n+tr\left(S\right)\right)/\left(n-2-tr\left(S\right)\right)$$

where $\hat{\sigma }$ is the estimated standard deviation of the error term and tr(S) is the trace of the projection matrix S (which directly maps the true value to the fitted value). In addition, the experiment uses the residual sum of squares (RSS) as an iterative convergence criterion.

#### 2.4.4. Characteristic Description and Statistics

Following the analytical framework in Figure 2, this article divides the independent variables into two categories: structure and neighborhood characteristics. The descriptions and statistics of the indicators are shown in Table 1. Structural characteristics are indicators that characterize the properties of housing units, including the usable area (AREA), orientation of windows (ORI), number of floors (FLOOR), building age (AGE), floor-area ratio (PR), green coverage rate (GR), and property fee (PF). Neighborhood characteristics describe residents’ accessibility to the urban infrastructure, including the road network distance to the nearest bus stations (BUS_D), entertainment facilities (ENT_D), hospitals (HOS_D), supermarkets (SOP_D), subway stations (SUB_D), large green spaces (GRE_D), and water bodies (WAT_D). In this paper, the natural logarithm of perceived greenery (LNPG) is used as a metric to characterize the adjacent greenery quality at the block level and introduced into the set of neighborhood characteristics

## 3. Results and Discussion

### 3.1. Distribution Map of Neighborhood-Level Perceived Greenery

**Figure 4:**
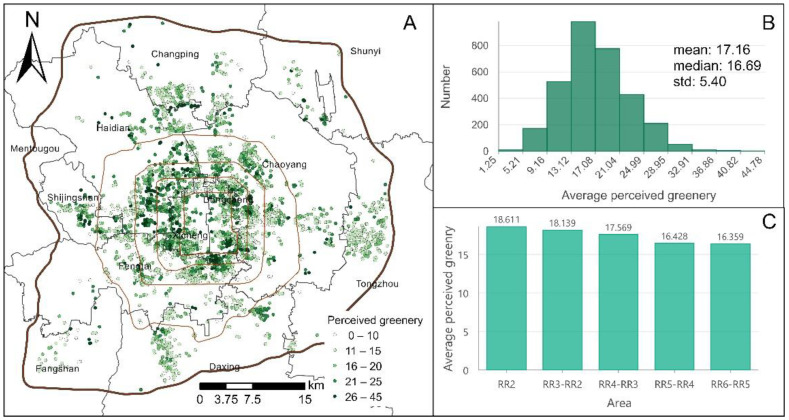
The perceived greenery distribution map and statistics in Beijing. Subplot (**A**): the perceived greenery distribution map. Subplot (**B**): the histogram and statistical results of average perceived greenery. The abbreviation “RR” in subplot (**C**) indicates ring roads, e.g., “RR3-RR2” indicates the Third to Second Ring Road areas.

The distribution map of residents’ perceived greenery at the neighborhood scale was drawn using ArcGIS (see Figure 4A). According to the quantile principle and a rounding up strategy, PG was divided into five intervals: 0–10 (very low), 11–15 (low), 16–20 (median), 21–25 (high), and 26–45 (very high). Figure 4B shows that the distribution of neighborhood-scale PG results is similar to a normal distribution, and the mean and median are very close (17.16 and 16.69). The average PG scores in the southern parts of the Xicheng District and the Dongcheng District, the southern part of the Haidian District, and the northern part of the Fengtai District exhibit high-value clusters. In Figure 4C, PG values exhibit a decreasing layout from the inside to outside areas of Beijing. Macroscopically, the PG scores in central urban areas (within the Fourth Ring Road) are relatively high. In summary, the neighborhood-scale PG results display obvious spatial variation, and the resident perceptions of greenery are unequal. Overall, there is considerable room for improvement in the PG level of housing units outside the Fourth Ring Road.

### 3.2. The Empirical Results and Hedonic Premium Analysis

According to the analytical framework used in this article (see Figure 2), we employed OLS, GWR, and MGWR to construct HPMs and compare the fitting performance, estimated parameters, and biases of different models. Then, the best-performing model was chosen to explain the marginal effects of characteristics in the study area. Finally, an analysis of the PG premium and its spatial patterns was performed.

#### 3.2.1. The Regression Result of the Global OLS Model

The coefficient of determination (R^2^) of the OLS model reached 0.65, and most variables were significant at the 0.01 level (see Table 2). Regarding structural characteristics, AREA, FLOOR, AGE, GR, and PF were significantly positively correlated with housing prices, and the results for AREA, FLOOR, and GR were consistent with those of a previous study [37], but the AGE and PF results were opposite. Notably, the OLS model ignores the estimation bias caused by spatial autocorrelation.

Among the neighborhood characteristics, BUS_D (0.17), WAT_D (0.06), and LNPG (0.11) were significantly positively correlated with housing prices. However, ENT_D (−0.08), EDU_D (−0.09), GRE_D (−0.01), and SUB_DIS (−0.07) displayed negative correlations. Compared with GRE_D, LNPG (0.105) made a larger positive contribution. In addition, LNPG displayed strong spatial autocorrelation (Moran’s I = 0.24, Z-value = 15.62). Therefore, to explain the spatial variations in coefficients, it is necessary to use spatial regression models for further discussion.

#### 3.2.2. Performance Comparison

By comparing the performance of Models 1 to 3 (see Table 2 and Table 3), we found that first, the coefficient of determination (0.802) of MGWR was better than that of GWR (0.782) and OLS (0.650), and the coefficients of both AICc and RSS were significantly lower. Second, the signs of the coefficients of GWR and MGWR were consistent, and the coefficients of the variables fluctuated slightly. The coefficients of LNPG in the GWR and MGWR models were 0.019 and 0.041, respectively, indicating that GWR underestimates the contribution of PG. Third, the standard error of the variables in MGWR was lower than that in GWR, and the coefficient estimates were comparatively more reliable. Fourth, there was a significant difference in bandwidth between the GWR and MGWR models. The optimal adaptive bandwidth of GWR was 360, which corresponds to a scale close to the block scale; the bandwidth of different variables in MGWR notably varied from 122 to 3175, corresponding to the local-to-global scales. Therefore, based on the global performance of the three models, we adopted the results of MGWR to further explain the characteristic premium.

#### 3.2.3. Explanation of Marginal Premiums

For the structural characteristics of Model 3 (see Table 3), increases in the AREA, FLOOR, GR, and PF of a unit can result in 0.8% (22,607 RMB), 0.4% (11,304 RMB), 0.3% (8478 RMB), and 2% (56,518 RMB) premium increases on average, respectively. The positive correlation between AREA and housing prices has been verified in other studies [54,55]. The FLOOR trend indicates that Beijing residents are more inclined to buy apartments in high-rise buildings because of their relatively low age and complete infrastructure. This result is similar to the findings of a case study in Hong Kong [56]. The variable GR reflects the green coverage within a housing estate, indicating that homebuyers prefer better living conditions with more greenery [38]. PF reflects the level of property service to a certain extent. The Beijing and Shanghai cases show that there is a significant positive correlation between PF and housing prices [38,39]. When housing in a community faces south (ORI = 1), the average housing price is 0.7% (19,781 RMB) higher than that for other orientations. AGE and PR are negatively correlated with housing prices, and unit increases in these variables cause prices to decrease by 0.2% (5652 RMB) and 0.9% (25,433 RMB), respectively. Thus, homebuyers are willing to pay for newer buildings and larger daily activity spaces. According to MGWR, the bandwidth of the model shows that AREA has the smallest influence scale, indicating that AREA fluctuates greatly in the study area, while other structural features are significant at the global level, indicating that they display spatial stability.

Of the neighborhood characteristics, BUS_D, ENT_D, HSP_D, and SOP_D were positively correlated with housing prices, which means that homebuyers are more inclined to buy houses far from these facilities because crowds and transportation facilities are associated with relatively high emissions and noise [8], which may reduce physical and mental health. In contrast, the housing prices of units close to schools, subway stations, and green spaces tended to be higher, and a 1 km decrease in distance to these facilities was associated with housing price increases of 2.8% (79,125 RMB), 1.9% (53,692 RMB), and 1.9% (53,692 RMB), respectively. This conclusion is consistent with those of previous studies [16,21,23]. The model results suggest that buyers have negative attitudes toward nearby water bodies. For every 1 km between a water body and a home, the average house price increases by 8.3% (234,550 RMB). This finding is contrary to that reported in a study by Luttik [57] in the Netherlands.

The spatial impact scale of neighborhood feature variables is heterogeneous. The accessibility bandwidths of ENT_D and WAT_D were 1735 and 1063, respectively, indicating that these spatial variations in characteristics are not significant. The bandwidth of EDU_D was small (519), which indicates that the parameter surface significantly changes in the study area. Because the educational resources in the Haidian District of Beijing far exceed those in other administrative regions, many Chinese families are willing to pay a high premium to purchase school district housing, improve the quality of education for their children, and enhance the competitiveness of future generations [58,59,60]. In addition, the bandwidths of other neighborhood characteristics reflect their relative spatial stability.

The coefficient of LNPG is estimated to be 0.041, and the corresponding economic premium (115,862 RMB) is much higher than those of GR (8478 RMB) and GRE_D (53,692 RMB). In addition, the bandwidth of LNPG (3172) indicates that the economic premium of perceived greenery is spatially stable. The development intensity of built-up areas in the study area is very high, but large-scale green parks and forests are relatively limited. The perceived greenery in residential areas mainly comes from the surrounding road greenery, corridors, and pocket parks. The relative scarcity of greenery resources has led to the urgent need for more perceivable greenery for the residents of Beijing.

### 3.3. The Spatial Patterns of Perceived Greenery Premiums

**Figure 5:**
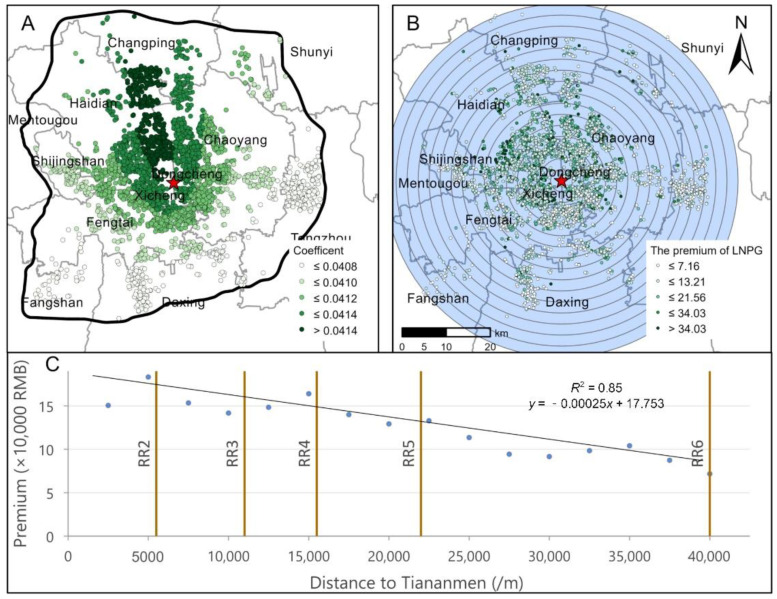
Spatial distribution of PG coefficients and premiums. Subplot (**A**) shows the distribution of PG coefficients. Subplot (**B**) takes Tiananmen as the center and uses an interval of 2.5 km to create a multiring buffer and show the PG premium gradient. The dotted brown line is the extension of Beijing’s east-west line. Subplot (**C**) is the premium gradient and the fitting line, and the solid brown line represents the reference positions of the five-ring roads, e.g., the abbreviation “RR2” represents the Second Ring Road.

We further visualized the coefficients and premiums of PG at the neighborhood scale to explore the corresponding spatial distribution patterns. The PG coefficients and their premium maps are shown in Figure 5.

Figure 5A depicts the coefficient surface of LNPG. All 3175 plots involved in the calculation were significant at the 0.01 level. The coefficient surface of PG reflects the clustering characteristics of high inside and low outside, as well as high in the north and low in the south; additionally, the overall spatial fluctuation is small (from 0.0408 to 0.0416). In the northwestern parts of the Chaoyang District and the Haidian District, high-value clusters radially extend outward, and low-value clusters appear in the southern and western areas near the Sixth Ring Road. The coefficient surface shows that homebuyer demands for perceived greenery exhibit spatial stationarity. Additionally, the overall perceived greenery premium rate is close to 4.1%.

The LNPG premium reflects the marginal price increase (/10,000 RMB) for every 1% change in the housing-scale PG. We found that the spatial distribution of PG premiums is unequal. Due to the large spatial variations in PG at the neighborhood level (Figure 3), when the marginal coefficient of PG is stable (Figure 5A), the economic premium displays obvious spatial heterogeneity. As shown by the multiring buffers and regression results (Figure 5B,C), the average premium of PG varies from 0.72 to 3.40 million RMB and displays a linear decrease from the inside to the outside of Beijing. The gradient fitting model of PG is close to *y* = −0.00025*x* + 17.753, and R^2^ reaches 0.85. This finding implies that for every 1 km increase in the Euclidean distance from a house to Tiananmen Square, the potential PG premium will decrease by approximately 2500 RMB on average. In addition, the PG premiums display very significant north-south spatial disparities (see Figure 5B). The average premium in the north is approximately 1.45 million RMB, and that in the south is approximately 1.07 million RMB. The neighborhood-scale PG and premium performance within the Fourth Ring Road (central city) are significantly better than those from the Fourth to Sixth Ring Roads (outer urban areas). Therefore, the housing-scale PG premiums are mainly restricted by the monocentric ring development pattern and relative central location.

### 3.4. Policy Recommendations

The overall PG at the neighborhood scale in Beijing displayed positive externalities. The quality of public green spaces in Beijing still has room to improve, especially in areas adjacent to residential blocks, as manifested by the low PG values of home buyers around such blocks. Our results show that greening services around neighborhoods in outer urban areas (especially those located in southern Beijing) lag behind those in developed and built-up urban areas. The supply capacity of greening services around residential blocks at the edge of the sixth ring is insufficient. The government should strengthen the establishment of vertical greenery in outer urban areas. For instance, trees with large canopies and narrow spacing can be planted to enhance the neighborhood-level PG. Improved PG in outer urban areas will result in two main benefits: it will help attract migrant populations to outer urban satellite towns, thus playing a certain role in decongesting the population in the central city, and it will provide greening resource equality and have positive effects on the overall ecological benefit and stability of the urban ecosystem.

From the residents’ perspective, home sellers are the direct beneficiaries of housing premiums (real estate appreciation). They not only enjoy greening services around housing units, but also receive premium benefits from them. Nevertheless, home buyers pay premium costs out of actual need. The government can levy an appropriate “greening premium fee” obtained from sellers when secondhand houses are sold because they acquire the utility and dividends of public green spaces but do not offer any direct maintenance costs. Through model 3 in this paper, the PG associated with each housing unit premium can be obtained for different locations based on the economic premium map (Figure 5B). Moreover, assuming that the green perception of all communities was to increase by one unit, the “greening premium fee” was unified at 0.02% of a home sale price, which could result in a total monetary premium exceeding 8.82 million RMB across the city. The government can coordinate the above premium funds and deploy them in future urban forestry and greening projects, thereby promoting equity in the development of neighborhood eco-environmental and economic benefits.

### 3.5. Limitations and Future Work

There is some room for improvement in this paper. First, in future studies, submarket HPM analysis can be performed for different school districts. Second, MGWR has high computational complexity and time costs, and the algorithm efficiency needs to be improved. In our experiment, it took nearly one hour to use the MGWR tool for one round of calculations (with an Intel i7-1065G7 CPU and 32 GB RAM). The current version of the MGWR tool struggles to run regional- and national-scale HPM models with massive records [52]. According to the road network topology, the road distance-based bandwidth could be added into the MGWR tool to improve realism. Finally, the analytical framework has large application potential, which can be deployed in other cities to analyze and compare the spatial patterns of housing premiums, thus providing an analytical pathway for assessing the connections between the economic benefits of real estate properties and public perceived greenery.

## 4. Conclusions

Public perceived greenery is an important influential factor that affects the health and sustainable development of urban residents. In this study, we provided a novel and comprehensive analytic framework coupling MGWR-HPM, multi-source geographic big data, and deep learning to explore the spatial impact of economic premiums at the neighborhood level. By conducting a comparison with the classic OLS and GWR model forms, we found that the MGWR-based HPM framework performs better in terms of fitting performance, the coefficient of determination, and error estimations. According to our analytical framework and empirical results, homebuyers in Beijing have a strong demand for neighborhood-level perceived greenery enhancement globally, and the average premium rate reached 4.1%. The perceived greenery premium displays an imbalanced distribution, which is close to linearly decreasing with north-high and south-low patterns. Specifically, for every 1 km increase in distance from the city center (Tiananmen), the perceived greenery premium decreases by approximately 2500 RMB on average. The neighborhood-level perceived greenery outside the Fourth Ring Road in Beijing and the premiums there have enormous development potential, which could increase additional economic benefits for sustainable greening optimization and government management. The results of this study can help the government assess the premium of neighborhood-level perceived greenery and coordinate the equitable development of the real estate economy and livable neighborhoods.

## Figures and Tables

**Table 1:** Characteristic indicators and basic statistics.

Category	Variables	Description	Mean	Standard Error
*Dependent variable*	LNHP	Log selling price in 10,000 RMB (Chinese currency, US $1 = RMB 6.497)	5.667	0.536
*Structure characteristics*	AREA	Average usable area in the home (m^2^)	88.450	46.84
	ORI	Dummy variable; 1 if the building windows face south	0.783	0.412
	FLOOR	Average number of floors in the building	11.539	11.861
	AGE	2021 minus the year of construction of the building	19.641	13.747
	PR	Floor-area ratio	2.524	1.549
	GR	Green coverage rate (%)	32.462	7.486
	PF	Property management fee (RMB/m^2^/ month)	1.764	1.419
*Neighborhood characteristics*	BUS_D	Road distance to the nearest bus station (km)	0.236	0.205
	ENT_D	Road distance to the nearest entertainment facility (km)	0.132	0.215
	HSP_D	Road distance to the nearest hospital (km)	0.182	0.219
	EDU_D	Road distance to the nearest school (km)	0.182	0.239
	SOP_D	Road distance to the nearest store (km)	0.097	0.172
	SUB_D	Road distance to the nearest subway station (km)	1.471	1.334
	GRE_D	Road distance to the nearest green space (km)	0.253	0.245
	WAT_D	Road distance to the nearest water body (km)	0.732	0.503
	LNPG	Logarithmic of average perceived greenery at the house level	2.798	0.337

**Table 2:** Performance of OLS regression (*n* = 3175).

Variables	Model 1: OLS Regression		
	Unstandardized Coefficients	Standard Error	*p*-Value
Constant	4.752 **	0.071	0.000
*Structure characteristics*			
AREA	0.007 **	0.000	0.000
ORI	0.012	0.017	0.484
FLOOR	0.004 **	0.001	0.000
AGE	0.001 **	0.001	0.001
PR	0.005	0.004	0.248
GR	0.003 *	0.001	0.003
PF	0.056 **	0.006	0.000
*Neighborhood characteristics*			
BUS_DIS	0.173 **	0.034	0.000
ENT_DIS	−0.075	0.040	0.061
HSP_DIS	−0.104 *	0.040	0.010
EDU_DIS	−0.091 *	0.031	0.004
SOP_DIS	−0.102	0.057	0.072
SUB_DIS	−0.069 **	0.005	0.000
GRE_DIS	−0.012 *	0.028	0.010
WAT_DIS	0.060 **	0.013	0.000
LNPG	0.105 **	0.020	0.000
R^2^	0.653		
Adjusted R^2^	0.650		
AICc	2723		
RSS	433		

** significant at the 1% level; * significant at the 5% level.

**Table 3:** Performance of GWR and MGWR (*n* = 3175).

	Model 2: GWR			Model 3: MGWR		
Variables	Unstandardized Coefficients (Mean)	Standard Error	Bandwidth	Unstandardized Coefficients (Mean)	Standard Error	Bandwidth
Constant	4.750 **	0.446	360	4.560 **	0.240	54
*Structure characteristics*						
AREA	0.008 **	0.001	-	0.008 **	0.001	122
ORI	0.073	0.005	-	0.007	0.001	3175
FLOOR	0.002 **	0.005	-	0.004 **	0.000	3175
AGE	−0.002 **	0.003	-	−0.002 **	0.000	3175
PR	−0.007	0.011	-	−0.009	0.000	3175
GR	0.002 **	0.004	-	0.003 **	0.000	3175
PF	0.044 **	0.037	-	0.020 **	0.000	3175
*Neighborhood characteristics*						
BUS_D	0.034 **	0.117	-	0.031 **	0.004	3172
ENT_D	0.045 *	0.219	-	0.062 *	0.056	1735
HSP_D	0.035 *	0.144	-	0.026 *	0.003	3175
EDU_D	−0.039 *	0.173	-	−0.028 *	0.003	519
SOP_D	0.038	0.235	-	0.028	0.009	3132
SUB_D	−0.016 **	0.052	-	−0.019 **	0.001	3175
GRE_D	−0.003 **	0.124	-	−0.019 **	0.003	3175
WAT_D	0.024 **	0.107	-	0.083 **	0.029	1063
LNPG	0.019 **	0.121	-	0.041 **	0.000	3175
R^2^	0.810			0.814		
Adjusted R^2^	0.782			0.802		
AICc	883			378		
RSS	185			179		

** significant at the 1% level; * significant at the 5% level (average performance).

## Data Availability

The Baidu Street View access instructions can be found at http://lbsyun.baidu.com/index.php?title=viewstatic (accessed on 12 August 2020).
